# PECAM/eGFP transgenic mice for monitoring of angiogenesis in health and disease

**DOI:** 10.1038/s41598-018-36039-2

**Published:** 2018-12-04

**Authors:** Florian Winkler, Katia Herz, Sarah Rieck, Kenichi Kimura, Tianyuan Hu, Wilhelm Röll, Michael Hesse, Bernd K. Fleischmann, Daniela Wenzel

**Affiliations:** 1 Institute of Physiology I, Life&Brain Center, Medical Faculty, University of Bonn, Bonn, Germany; 2 Department of Cardiac Surgery, Medical Faculty, University of Bonn, Bonn, Germany

## Abstract

For the monitoring of vascular growth as well as adaptive or therapeutic (re)vascularization endothelial-specific reporter mouse models are valuable tools. However, currently available mouse models have limitations, because not all endothelial cells express the reporter in all developmental stages. We have generated PECAM/eGFP embryonic stem (ES) cell and mouse lines where the reporter gene labels PECAM^+^ endothelial cells and vessels with high specificity. Native eGFP expression and PECAM staining were highly co-localized in vessels of various organs at embryonic stages E9.5, E15.5 and in adult mice. Expression was found in large and small arteries, capillaries and in veins but not in lymphatic vessels. Also in the bone marrow arteries and sinusoidal vessel were labeled, moreover, we could detect eGFP in some CD45^+^ hematopoietic cells. We also demonstrate that this labeling is very useful to monitor sprouting in an aortic ring assay as well as vascular remodeling in a murine injury model of myocardial infarction. Thus, PECAM/eGFP transgenic ES cells and mice greatly facilitate the monitoring and quantification of endothelial cells *ex vivo* and *in vivo* during development and injury.

## Introduction

Vascular growth is a key process during development and it also determines vessel remodeling in response to injury. Therefore transgenic models, in which endothelial cells are specifically labeled are valuable tools to monitor vascular network formation and to generate novel strategies for (re)vascularization. In the past different potentially endothelial-selective reporter mouse lines (tie1/eGFP^1^, tie2/eGFP^2^, flk1/eGFP^3^, flt1/eGFP^4^) have been generated. However, their use is limited because they either show expression restricted to distinct developmental stages or they do not express the reporter in all endothelial cells or endothelial cells of all organs^1,2,4^. The respective Cre mice often displayed expression in non-endothelial cells mostly because the promoters are also active in other cell types during development^5–9^. To overcome these limitations and to generate a reporter gene model with endothelial-specific expression at all stages and in all vascular beds we have chosen the promoter of the platelet endothelial cell adhesion molecule 1 (PECAM) driving the eGFP reporter gene. PECAM is a cellular adhesion and signaling receptor, which acts as a mechanosensor and plays a major role during extravasation of leukocytes and the regulation of the vascular permeability barrier^10,11^. Because of the particularly strong expression on the endothelium PECAM is considered as the classical endothelial marker. We therefore thought that the PECAM promoter driving a reporter gene should be ideal for robust endothelial cell labeling and also for the monitoring of cell fate and vascular remodeling in injury models *in vivo*. Herein, we have generated PECAM/eGFP embryonic stem (ES) cells and the respective mouse line. This allowed direct visualization of the endothelium in all vascular beds at single cell resolution. Furthermore, we demonstrate the utility of the PECAM/eGFP mouse model to investigate angiogenesis in aortic outgrowth experiments and to monitor vascularization in the heart after experimental myocardial infarction.

## Methods

### Generation of PECAM/eGFP BAC vector

For the generation of the PECAM/eGFP vector, the 220 kbp bacterial artificial chromosome (BAC) RP23-120D9 (obtained by the Children’s Hospital Oakland Research Institute, CHORI) covering the promoter region of the PECAM gene was transferred into SW105 E. coli cells by electroporation. This procedure we have reported recently^4^. Briefly, an eGFP-pA cDNA was created for insertion at the initiation codon in the first exon of the PECAM gene within the BAC. Homologous arms were generated by PCR using the pEGFP-1 vector as template. For this purpose the following primers were used: (fw: 5′GCA GAA GTC TTT CAG GAT TCA GCT GAG GTG GGC CTC AGT CGG CAG ACA AGT GGC CAC AAC CAT GGT GAG 3′, rev: 5′ AAG AAG GGA AGA AGG AAG CAG GTT AGA AAC TCA CAG AGC ACC AGC GTG AGC CCG CG TTT ATG AAC AAA CGA C 3′). Electrocompetent SW105 E.coli cells containing the BAC were then electroporated with the targeting cassette and clones with successful recombination were selected with kanamycin and chloramphenicol. Homologous recombination was verified by PCR with primers spanning the homology region. Then G4 ES cells were transfected with the PECAM/eGFP BAC.

### PECAM/eGFP ES cells

In order to generate transgenic ES cell clones 5 × 10^6^ G4 murine ES cells were electroporated with 40 µg of linearized PECAM/eGFP vector with one pulse at 250 V and 500 µF (Bio-Rad Gene Pulser, Munich, Germany). ES cells were then plated on 10 cm cell culture dishes covered by feeder cells in Dulbecco’s modified Eagle medium (DMEM, Life technologies, Darmstadt, Germany), supplemented with 15% fetal calf serum (FCS), 0.1 mM nonessential amino acids, 2.0 mM L-glutamine, 50 µg/ml penicillin and streptomycin (all from Invitrogen, Karlsruhe, Germany), 0.1 mM β-mercaptoethanol (Sigma Aldrich, Steinheim, Germany), 500 U/ml leukemia inhibitory factor (LIF, Chemicon, Hofheim, Germany). Selection was performed using 165 µg/ml G148 (Invitrogen, Karlsruhe, Germany). Resistant colonies were then picked and propagated for further differentiation which was performed using embryoid body (EB) formation via mass culture^12^ or hanging drop method^13,14^. Therefore, ES cells were cultivated in Iscove’s modified Dulbecco’s medium (IMDM) (Invitrogen, Karlsruhe, Germany) supplemented with 20% FCS plus the additives mentioned above in the absence of LIF. After 10 days of differentiation, transgenic EBs were plated on cover slips coated with 0.1% gelatine solution (Sigma Aldrich, Steinheim, Germany).

### PECAM/eGFP transgenic mice

G4 PECAM/eGFP ES cell clones, which were screened for normal 40 chromosome karyotype, were used for aggregation with diploid CD1 embryos at morula stage as described previously^15^. Chimeric mice could be identified due to their spotted coat and were bred with CD1 mice to examine germline transmission. PCR of tail tips revealed the transgenic offspring: Following primers were used (fw: 5′ ACC TGC CAT CAC GAG ATT TC 3′, rev: 5′ TTC TCC TAC ACC GCT GTC TC 3′). The expression of the transgene was studied at day 9.5 and 15.5 of embryonic development (E9.5, E15.5) as well as in adult organs and tissues by fluorescence microscopy (AxioZoom V16, AxioObserver Z1 and Axiovert 200 M, Carl Zeiss, Oberkochen, Germany).

### Histology and immunohistochemisty

For generation of cryosections adult hearts were perfused with PBS followed by 12 ml 4% paraformaldehyde (PFA) in phosphate buffered saline (PBS) at room temperature (RT). E9.5 embryos and adult vessels were incubated for only 2 hours with 4% PFA. Adult tissues and E15.5 embryos were fixated over night at 4 °C. Soft tissues were dehydrated with 20% sucrose in PBS over night before they were embedded in OCT compound (Tissue-Tek, Sakura Finetek Europe B.V., Alphen aan den Rijn, Netherlands). Then 10 µm thick sections were generated with a cryotome (CM 3050 S, Leica Microsystems, Wetzlar, Germany)^16,17^. Cells and soft tissues were permeabilized using 0.2% Triton-X100 (Sigma Aldrich, Steinheim, Germany). Tibial bones were fixed in 4% PFA overnight, before being incubated in 10% (4 hours) and 20% (overnight) sucrose for cryopreservation. Then they were embedded in SCEM (Section-Lab Co. Ltd., Hiroshima, Japan) and sectioned into 5 µm thick slices using the cryostat mentioned above according to the Kawamoto method^18^. Permeabilization of bone tissue was performed using PBS containing 0.1% Tween 20 (AppliChem GmbH, Darmstadt, Germany). In all tissues unspecific binding sites were blocked by adding 5% donkey serum (Jackson Immuno Research, Suffolk, Great Britain) in PBS for 30 minutes. Immunostainings were performed as described recently^19,20^. Sections as well as fixated cells were stained with primary antibodies against PECAM (1:800, BD Biosciences, Heidelberg, Germany), *Griffonia Simplicifolia* lectin 1 (Vector Laboratories, Burlingame, USA), CD45 (1:800, Merck Millipore, Billerica, USA), endomucin (EMCN, 1:200, R&D Systems, Minneapolis, USA) or LYVE 1 (1:100, ReliaTech, Wolfenbüttel, Germany). To visualize primary antibodies and nuclei Cy3 or Cy5 anti-rat, anti-goat or anti-rabbit antibodies (all 1:400, Jackson Immuno Research, Suffolk, Great Britain) and Hoechst 33342 (1 mg/ml, Sigma-Aldrich, Taufkirchen, Germany), respectively, were applied for 1 hour at RT. Pictures of stained sections of cells and soft tissues were taken with an AxioObserver Z1 and Axiovert 200 M microscope with ApoTome optical sectioning module (Carl Zeiss, Oberkochen, Germany). Fluorescence images of tibial sections were acquired using a confocal microscope (ECLIPSE Ti, Nikon, Tokio, Japan). In adult hearts (n = 3) eGFP^+^ and PECAM^+^ endothelial cells were quantified in 5 transversal sections per heart.

### Isolation of adult cardiac endothelial cells via magnet-activated cell sorting (MACS)

MACS was performed as described before^21^. 6- to 8-week-old WT and PECAM/eGFP mice were sacrificed and hearts were isolated. Then, the aorta was perfused with 1.5 ml PBS before removing the atria. Ventricles were minced and enzymatically dissociated at 37 °C and 800 rpm for 25 minutes. For one heart 800 µl of enzyme buffer was used. To prepare the enzyme puffer 5 mg liberase III (Roche, Mannheim, Germany) was dissolved in 20 ml 10 mM HEPES solution in HBSS (Ca^2+^, Mg^2+^) and aliquots of 800 µl were stored at −20 °C. Before the dissociation of tissue 1 µl 100 U/ml DNAse I (Life technologies, Darmstadt, Germany) was applied to the enzyme buffer aliquot. After the first dissociation the supernatant was removed and stored on ice while the remaining tissue was used for dissociation again. Cells in suspension were magnetically labelled and separated using PECAM microbeads (Miltenyi Biotec, Bergisch Gladbach, Germany) according to the manufacturer’s instructions. Purity was examined by flow cytometric quantitation of eGFP^+^/PECAM-PE^+^ (BD Biosciences, Heidelberg, Germany) cell populations using a CyFlow space flow cytometer (Sysmex Partec, Görlitz, Germany).

### Western blot analysis

For protein isolation cells were lysed in RIPA buffer (2 mM EDTA, 25 mM Tris-HCl, 150 mM NaCl, 0.1% (w/v) sodium deoxycholat, 0.1% (w/v) sodium dodecyl sulfate, 1% (v/v) Nonidet^TM^ P40) containing protease inhibitors (Roche) directly after MACS procedure. The protein concentration was determined using the Pierce BCA Protein Assay Kit (Thermo Fischer Scientific). SDS-PAGE was performed in a 10% polyacrylamide gel. Afterwards, proteins were transferred to a nitrocellulose membrane using the semi-dry blotting procedure. Following primary antibodies were applied: anti-GFP (mouse, 1:1,000, Clontech), peroxidase-coupled anti-β-actin (1:10,000, Sigma Aldrich). A peroxidase-coupled anti-mouse antibody (1:10,000, Jackson ImmunoResearch) was applied as secondary antibody for eGFP detection. Chemiluminescence signals were detected using the SuperSignal^TM^ West Pico Plus Substrate (Thermo Fischer Scientific) and a ChemiDoc^TM^ MP imaging system (BioRad).

### Mouse aortic ring assay

Aortic ring assay was performed as described before^22^. 6-month-old PECAM/eGFP mice were killed and thoracotomy was performed to expose and dissect the thoracic aorta. The aorta was immediately transferred to a cell culture dish filled with Opti MEM (Life technologies, Darmstadt, Germany) and the peri-aortic fibroadipose tissue was removed carefully. One-millimeter-long aortic rings were sectioned and starved in Opti MEM with 1% penicillin and streptomycin over night at 37 °C. Then the aortic rings were embedded in 250 µl collagen gel per well in an 8 well µ-Slide (ibidi GmbH, Martinsried, Germany). This gel was generated by mixing 1.7 ml DMEM (Invitrogen, Karlsruhe, Germany) with 600 µl 3.79 mg/ml collagen I (Merck Millipore, Billerica, USA) and 2 µl 5 M NaOH. Polymerization of the collagen gels was achieved by incubation at RT for 15 minutes and at 37 °C for 60 minutes. The polymerized gels containing the aortic rings were covered with 180 µl Opti-MEM containing 1% penicillin and streptomycin and 2% FCS which was exchanged every third day. Cultures were kept at 37 °C and the explants were monitored for the outgrowth of endothelial cells on a daily basis using a stereomicroscope (AxioZoom V16, Carl Zeiss, Oberkochen, Germany).

### Experimental myocardial cryoinfarction of PECAM/eGFP mice

Animals experiments were approved by the local ethics committee and carried out according to the guidelines of the German law of protection of animal life with approval by the local government authorities (Landesamt für Natur, Umwelt und Verbraucherschutz Nordrhein-Westfalen (LANUV), NRW, Germany) and in accordance with the ethical standards laid down in the 1964 Declaration of Helsinki and its later amendments. Myocardial cryoinfarctions were generated as described before^17^. In order to examine the vascularization of the (peri-) infarct zone mice were sacrificed 3 or 6 days after the cryolesion. Hearts were harvested and immunohistochemical processing and staining was performed as explained above.

## Results

### Generation of PECAM/eGFP embryonic stem (ES) cells

**Figure 1:**
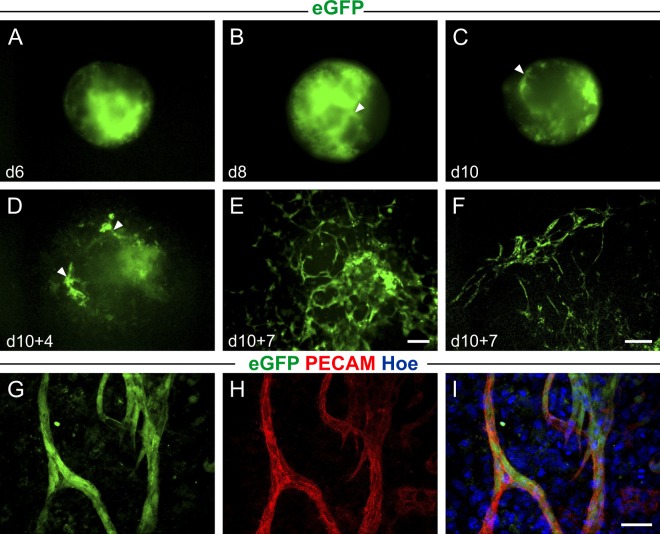
Endothelial-specific eGFP expression in embryoid bodies (EBs) derived from PECAM/eGFP ES cells. (**A**–**C**) Fluorescence images show eGFP^+^ clusters at day 6 (**A**) and early vascular network formation in EBs at day 8 (**B**) and day 10 (**C**) of suspension culture. Arrow heads indicate eGFP^+^ sprouts. (**D**–**F**) In plated EBs distinct sprouts can be observed at day 10 + 4 (**D**) resulting in a complex vascular network in the center (**E**) and the periphery (**F**) of the EB at day 10 + 7. (**G**–**H**) Immunofluorescence stainings of vascular networks reveal the co-localization of native eGFP and PECAM expression. Green = native eGFP, red = PECAM, blue = Hoechst; bars = 200 µm (**A**–**E**), 100 µm (**F**), 50 µm (**G**–**I**).

To generate a reporter gene construct that labels PECAM^+^ endothelial cells an eGFP expression construct was inserted into the first exon of a BAC spanning the murine PECAM promoter (Fig. S1). First, the expression pattern and specificity of the labeling were analyzed in murine ES cells. Therefore, the PECAM/eGFP vector was used for electroporation in G4 ES cells. As previously described for PECAM^+^ ES cells^23^, PECAM/eGFP^+^ cells were initially arranged in clusters (Fig. 1A). During differentiation in embryoid bodies (EBs) first eGFP^+^ sprouts appeared around d 8–10 (Fig. 1B,C, arrow heads). After plating of the EBs on d 10 eGFP^+^ networks developed through d 10 + 4 to d10 + 7 (Fig. 1D–F). Immunohistochemistry revealed that native eGFP expression of the sprouts (green, Fig. 1G,I) perfectly co-localized with PECAM staining (red, Fig. 1H,I), which confirms PECAM-specific expression of the reporter gene in the ES cell system.

### Characterization of PECAM/eGFP transgenic mouse embryos

**Figure 2:**
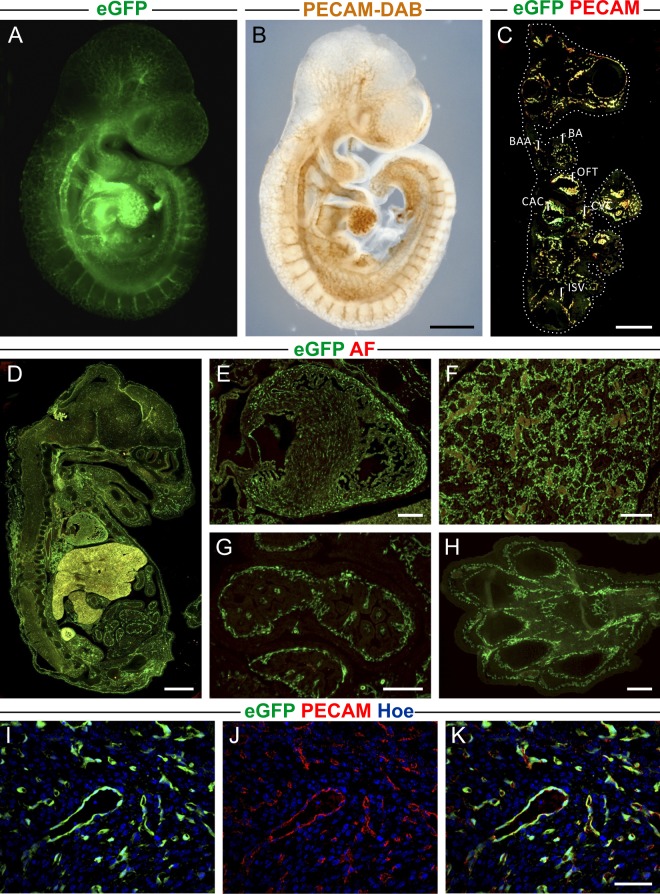
EGFP expression in PECAM/eGFP embryos at different developmental stages. (**A**) Fluorescence picture demonstrates bright native eGFP expression in network-like structures of embryos at E9.5. (**B**) PECAM-DAB whole mount staining of littermates shows a good overlap with eGFP^+^ structures in (**A**) confirming PECAM-specific eGFP expression. (**C**) In cryosections of E9.5 embryos co-localization of native eGFP (green) and PECAM staining (red) was observed. Strong eGFP (green) and PECAM (red) expression were identified in the branchial arch (BA), branchial arch artery (BAA), outflow tract of the heart (OFT), common atrial chamber (CAC), common ventricular chamber (CVC) as well as in intersomitic vessels (ISV). The dotted white line defines the surface of the embryo. (**D**–**H**) Sections of embryos at E15.5 reveal a strong eGFP expression in the developing vasculature (**D**), in particular in the heart (**E**), the lung **(F**), the gut (**G**), and the paw (**H**). (**I**–**K**) Immunofluorescence stainings of heart sections derived from a E15.5 embryo confirms PECAM-specific eGFP expression in blood vessels. Green = native eGFP, brown = PECAM-DAB, red = PECAM (**C**,**J**,**K**), autofluorescence (AF) (**D**–**H**), blue = Hoechst; bars = 500 µm (**A**–**C**), 100 µm (**D**), 200 µm (**E**–**H**), 50 µm (**I**–**K**).

Next, we generated a transgenic mouse line by aggregation of PECAM/eGFP ES cells with morula stage wildtype embryos. The mice were developmentally normal and no adverse effects of PECAM/eGFP expression could be detected. When transgenic E9.5 embryos were compared with littermate controls PECAM/eGFP^+^ embryos demonstrated eGFP^+^ developing vascular networks, while controls showed no fluorescence (Fig. S2A). In particular, bright sprouts were found in the brain (Fig. S2B), the heart (Fig. S2C) and the caudal region of the embryo (Fig. S2D) The eGFP fluorescence in the E9.5 embryo showed an excellent overlap with PECAM whole mount stainings (Fig. 2A,B). This could be also confirmed in cryosections where native eGFP (green) was co-localized with PECAM staining (red) throughout the embryo (Fig. 2C).

The strong eGFP labeling of the vasculature was also observed in cryosections derived from E15.5 embryos (Fig. 2D). At this stage, in particular highly vascularized organs such as the heart (Fig. 2E), lung (Fig. 2F), gut (Fig. 2G) and the paw (Fig. 2H) displayed bright native eGFP fluorescence (green). Also in these organs native eGFP expression and PECAM staining (red) showed a good overlap in cryosections (Fig. S2E–P). At high resolution immunostainings of heart sections demonstrated that almost all eGFP^+^ cells were also labeled with PECAM antibody (Fig. 2I–K) further confirming endothelial specific eGFP expression at E15.5.

### Analysis of PECAM/eGFP adult mice

**Figure 3:**
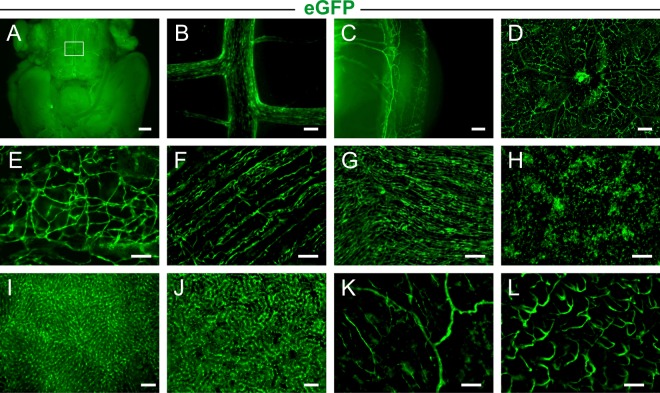
EGFP expression in organs of PECAM/eGFP adult mice. (**A–L)** Macroscopic images of organs from adult PECAM/eGFP mice illustrate strong native eGFP expression in network-like structures in the brain (**A**), the white rectangle in (**A**) defines the basilar artery shown in a close up in (**B**). In the eye, limbal vessels in the transition zone between cornea and sclera (**C**) as well as retinal vessels (**D**) exhibit a strong eGFP signal. EGFP expression was also found in the skin (**E**), the skeletal muscle (**F**), heart (**G**), lung (**H**), liver (**I**), kidney (**J**), gut (**K**) and uterus (**L**). Green = native eGFP; bars = 1000 µm (**A**), 100 µm (**B**,**E**–**L**), 200 µm (**C**,**D**).

**Figure 4:**
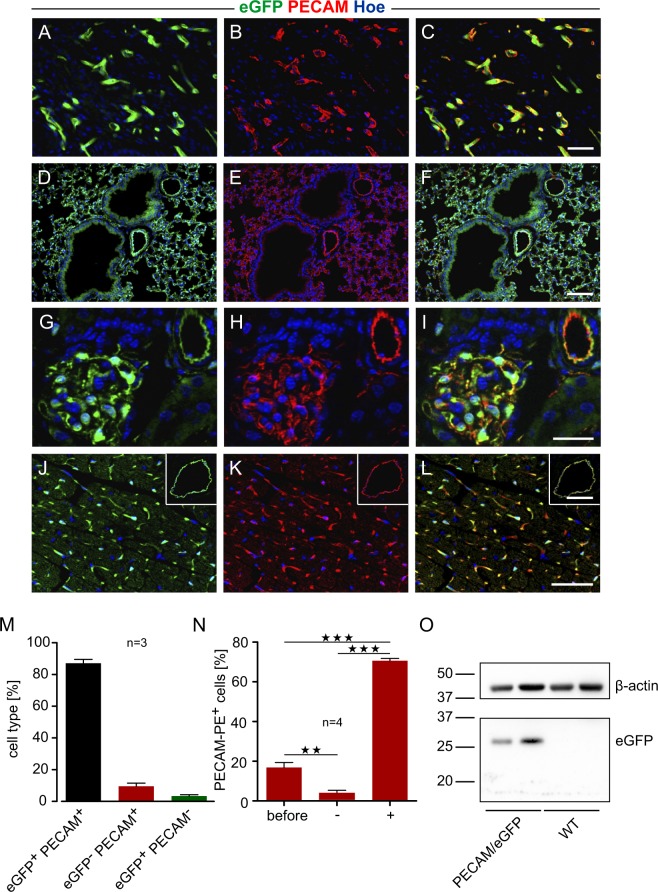
PECAM-specific eGFP expression in different organs of PECAM/eGFP adult mice. (**A**–**L**) Immunofluorescence stainings with an anti-PECAM antibody reveal prominent PECAM-specific native eGFP expression in the endothelium of the vasculature in the skin (**A**–**C**), in the lung (note that airway epithelium is eGFP^−^, (**D**–**F**), in the kidney (glomeruli and straight vessels, (**G**–**I**) and the heart (**J–L**). Co-localization of native eGFP fluorescence and PECAM staining was also found in endothelial cells of larger coronary arteries (inset, **L**). (**M**) Statistical analysis of eGFP and PECAM signals in myocardial sections shows co-localization in cardiac endothelial cells. (**N**) Flow cytometry analysis of PECAM-PE^+^ cells after MACS reveals successful purification of endothelial cells from adult hearts (before: before sorting, −: negative fraction, + : positive fraction). (**O**) Western blot analysis of these isolated PECAM^+^ cells confirms eGFP expression in PECAM/eGFP mice but not in WT mice (the membrane was cut at about 37 kDa to enable incubation with the two different antibodies against β-actin and eGFP), **p < 0.01, ***p < 0.001 (One way ANOVA, Tukey’s post hoc test, (N). Green = native eGFP, red = PECAM, blue = Hoechst; bars = 50 µm (**A–F**, **J–L**), 25 µm (**G**–**I**).

Then, we analyzed the expression pattern of the reporter gene in adult animals. Stereomicroscopy of adult mouse organs revealed that native eGFP expression was restricted to vascular network-like structures in all organs examined, such as the brain (Fig. 3A) and basilar artery (Fig. 3B), the outer eye (Fig. 3C) and retina (Fig. 3D), skin (Fig. 3E), skeletal muscle (Fig. 3F), heart (Fig. 3G), lung (Fig. 3H), liver (Fig. 3I), kidney (Fig. 3J), gut (Fig. 3K) and uterus (Fig. 3L). Immunostainings of cryosections using anti-PECAM antibody demonstrated that cutaneous vessels (Fig. 4A–C), the alveolar capillary network and peribronchial vessels (Fig. 4D–F), glomeruli and interlobular vessels of the kidney (Fig. 4G–I), vessels of the uterus (Fig. S3A–C) and gut (Fig. S3D–F) as well as cardiac capillaries (Fig. 4J–L) and coronary arteries (Fig. 4J–L, inset) displayed strong eGFP fluorescence and revealed almost a complete overlap with PECAM staining underscoring endothelial cell-specific eGFP expression. Within the heart, we analyzed eGFP expression in detail by cell counting in sections and found that 87.1 ± 0.02% of the labeled cells were eGFP^+^PECAM^+^, 9.5 ± 0.01% eGFP^−^PECAM^+^ and only 3.4 ± 0.0% appeared to be eGFP^+^PECAM^−^ (n = 3, Fig. 4M). Hence eGFP labeling in PECAM/eGFP adult hearts is highly specific and the vast majority of PECAM^+^ cells show eGFP fluorescence. To analyze PECAM and eGFP expression in isolated endothelial cells, we have performed magnet-activated cell sorting (MACS) of adult hearts derived from WT and PECAM/eGFP mice using an anti-PECAM antibody. Flow cytometry analysis with anti-PECAM-PE antibody staining demonstrated successful purification. (Fig. 4N). Western blot analysis of the PECAM^+^ cells then further proved eGFP expression of PECAM^+^ cells in PECAM/eGFP animals while there was no signal in WT animals confirming specifity of the eGFP antibody (Fig. 4O).

Next, we also investigated the eGFP expression in large arteries and veins. While stereomicroscopy revealed that in our previously generated flt-1/eGFP mouse model eGFP expression was absent in large arteries^4^ (Fig. S4A–E), in the PECAM/eGFP mouse line we detected prominent eGFP expression (Fig. S4A) in the endothelium of aorta (Fig. S4F–I) and carotid arteries. Similarly, also in large veins such as the portal vein we found a complete overlap of eGFP expression and PECAM staining (Fig. S4J–L).

**Figure 5:**
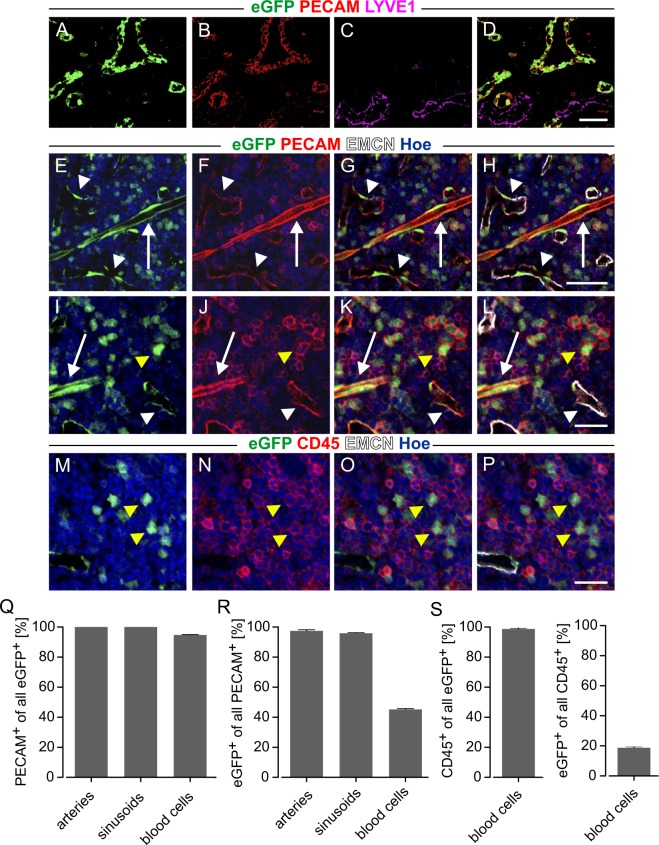
EGFP expression in lymphatic tissue and bone marrow. (**A**–**D**) Sections of PECAM/eGFP lymph node. Native eGFP expression (**A**) shows a good overlap with PECAM staining (**B**), whereas lymphatic vessels stained with LYVE1 (**C**) are eGFP^−^. (**E**–**P**) Immunofluorescence stainings of bone marrow tissue. (**E**–**L**) Native eGFP is strongly co-localized with PECAM^+^EMCN^−^ arteries (white arrows) and PECAM^+^EMCN^+^ sinusoids (white arrowheads). (**I**–**P**) Roundish eGFP^+^ cells can be identified as blood cells by their strong PECAM and CD45 signal (yellow arrowheads). (**Q**–**S**) Statistical analysis of eGFP, PECAM and CD45 expression in arteries, sinusoids and blood cells. Green = native eGFP, red = PECAM (**B**,**D**,**F**–**H**,**J**–**L**,), CD45 (**N**–**P**), purple = LYVE1, white = EMCN, blue = Hoechst; bars = 50 µm (**A–H**), 25 µm (**I–P**).

Besides expression in endothelial cells PECAM can be also found in hematopoietic cells. We therefore characterized PECAM/eGFP expression in these cells by generating cryosections of lymph nodes and tibial bone marrow. Native eGFP fluorescence was identified in PECAM^+^ blood vessels in lymph nodes (Fig. 5A,B,D), whereas no signal was detected in LYVE1^+^ lymphatic vessels (Fig. 5C,D). In the bone marrow immunofluorescence stainings illustrated that eGFP was expressed by elongated PECAM^+^EMCN^-^ arteries (white arrows) and PECAM^+^EMCN^+^ sinusoids (white arrowheads) (Fig. 5E–L). Moreover, there were also some roundish eGFP^+^ cells expressing PECAM (yellow arrowheads) (Fig. 5I–L) or CD45 (yellow arrowheads) (Fig. 5M–P), which is indicative of hematopoietic cells. Quantitative analysis demonstrated that also in the bone marrow eGFP expression was highly specific for PECAM^+^ arteries, sinusoids and blood cells (arteries 100.0 ± 0%, sinusoids 100.0 ± 0%, blood cells 94.8 ± 0.0%, n = 4, PECAM^+^ of all eGFP^+^, Fig. 5Q). Moreover, almost all of the PECAM^+^ cells in arteries and sinusoids were labeled by eGFP (arteries 97.5 ± 0.02%, n = 4, sinusoids 95.9 ± 0.0%, n = 4), but only about 40% of the PECAM^+^ blood cells displayed eGFP expression (45.2 ± 0.0%, n = 4, Fig. 5R). Almost all of these eGFP^+^ blood cells also expressed the pan hematopoietic marker CD45 (98.8 ± 0.0%, n = 4), while only about 20% of all CD45^+^ cells were labeled by eGFP (18.5 ± 0.0%, n = 4, Fig. 5S). This indicates that only a part of the hematopoietic cells is PECAM^+^ and signals are very low so that eGFP labeling is difficult to detect in these cells.

### PECAM/eGFP mice for aortic ring assay and experimental myocardial cryoinfarction

**Figure 6:**
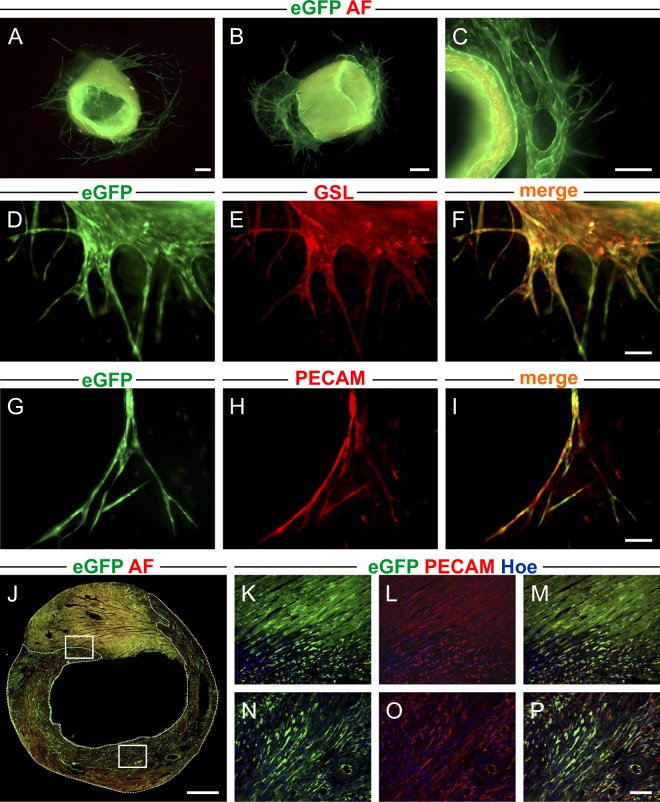
Utility of PECAM/eGFP mice for analysis of angiogenesis assays and *in vivo* injury model. (**A**–**C**) PECAM/eGFP aortic rings demonstrate eGFP^+^ sprouts in a collagen gel assay at d8 (A, transversal view), (**B**, frontal view) and d10 (**C**, higher magnification). (**D**–**I**) *Griffonia Simplicifolia Lectin* (red, **D**–**F**) and PECAM stainings (red, **G–I**) of aortic sprouts confirm the endothelial character of eGFP^+^ elongated structures. (**J**) Fluorescence picture 3d after myocardial cryoinfarction in PECAM/eGFP mice shows native myocardium with high autofluorescence (**J**, top) and the infarcted area with low background fluorescence (**J**, bottom). (**K**–**P**) PECAM staining (red) confirms endothelial-specific eGFP expression in the capillaries of the border zone (**K**–**M**) and the center (**N**–**P**) of the cryoinfarction. The white rectangles in (**J**) define magnified regions of consecutive cryosections shown in (**K**–**M**) and (**N**–**P**). Green = native eGFP, red = autofluorescence (AF) (**A**–**C**,**J**), GSL (**E**,**F**), PECAM (**H,I,L,M,O,P**), blue = Hoechst; bars = 200 µm (**A**–**C**), 100 µm (**D**–**F**, **G**–**I**), 500 µm (**J**), 50 µm (**K**–**P**).

To determine if the PECAM/eGFP mouse line can be also used in order to facilitate analysis of standard angiogenesis assays we isolated aortas and performed an aortic ring sprouting assay. For this purpose PECAM/eGFP aortic rings were embedded in a collagen matrix and sprouts could be easily visualized by native eGFP fluorescence at d8 (Fig. 6A,B) and d10 (Fig. 6C). Immunohistochemical analysis with *Griffonia Simplicifolia* lectin (GSL, Fig. 6D–F) and PECAM (Fig. 6G–I) staining confirmed the endothelial character of these eGFP^+^ sprouts.

Additionally, we also tested the PECAM/eGFP mouse line in an injury model *in vivo*, because in particular the processing and analysis of revascularization of infarcted heart tissue can be difficult. This is due to staining artefacts, as antibody staining in the infarct area often lacks specificity^24^. Therefore, myocardial cryoinfarctions were generated in PECAM/eGFP mice and vascularization was analyzed on d 3. In the infarction area background fluorescence was strongly decreased (area surrounded by dotted line, Fig. 6J) and in the overview many eGFP^+^ elongated structures could be identified. Immunostainings of cryosections revealed that there was a good overlap of eGFP fluorescence (green) and PECAM staining (red) in the border zone (upper rectangle Fig. 6J, Fig. 6K–M) and also in the center of the cryoinfarction (lower rectangle Fig. 6J, Fig. 6N–P). Thus, the PECAM/eGFP mouse line enables straightforward identification of endothelial structures in *ex vivo* angiogenesis assays and injury models *in vivo* without any additional processing and staining procedures.

## Discussion

Herein, we have generated a PECAM/eGFP murine ES cell and a transgenic mouse model for live monitoring of endothelial cells in small and large vessels *in vitro*, *ex* and *in vivo*.

For the generation of transgenic models we have chosen the PECAM promotor driving the reporter gene eGFP. During development the intraembryonic PECAM expression already starts at 7.8 dpc^25^ and persists throughout adulthood in the entire vascular endothelium^26^. PECAM/eGFP plasmids had been used for *in vitro* transfection of ES cells^27–29^ and HUVECs^30^ before, however, the generation of a PECAM/eGFP transgenic mouse has not been reported so far. In our PECAM/eGFP ES cell and mouse lines we have found endothelial-specific expression of eGFP at all stages and in vessels of all calibers. This expression pattern, which recapitulates the endogenous PECAM expression, is probably due to the inclusion of all regulatory sequences in the PECAM BAC that was used as transgenic vector. Apart from endothelial cells, PECAM expression can be also found on the surface of platelets and leukocytes, however, in these cells expression is described to be much lower^31^. This is in accordance with our data, as only a small number of eGFP^+^/PECAM^+^ hematopoietic cells was found in the bone marrow and this is most likely due to the overall low transgene expression in these cells. All our experimental data underscore the high specificity of the transgene expression for endothelial and also hematopoietic cells.

We are convinced that the high specificity of the labeling is very helpful for the analysis of state of the art *ex vivo* angiogenesis assays, such as the aortic ring assay^32^. In fact fluorescence microscopy enabled us to clearly distinguish between eGFP^+^ endothelial cells and other types of sprouting cells (i.e. fibroblasts and smooth muscle cells), thereby avoiding additional immunostainings, which may also facilitate the quantification of results^33^. Moreover, the PECAM/eGFP mouse model is helpful for the monitoring of revascularization processes upon injury (e.g. myocardial infarction). Currently, fixation and antibody staining is required, which adds a confounding factor, as unspecific stainings in injured tissues make it difficult to discern real signals from artefacts^24^. Apart from tracking endothelial cells *in situ* the PECAM/eGFP model can be also used as a source of *in vivo* labeled endothelial cells for cell transplantation or replacement experiments. These strategies are currently extensively investigated for the treatment of ischemia or endothelial cell damage.

## Electronic supplementary material

Data supplement

## Data Availability

Data are available upon request.

## References

[1] Iljin K (2002). A fluorescent Tie1 reporter allows monitoring of vascular development and endothelial cell isolation from transgenic mouse embryos. FASEB J.

[2] Motoike T (2000). Universal GFP reporter for the study of vascular development. Genesis.

[3] Ishitobi H (2010). Flk1-GFP BAC Tg mice: an animal model for the study of blood vessel development. Exp Anim.

[4] Herz K (2012). Live monitoring of small vessels during development and disease using the flt-1 promoter element. Basic Res Cardiol.

[5] Terry RW, Kwee L, Baldwin HS, Labow MA (1997). Cre-mediated generation of a VCAM-1 null allele in transgenic mice. Transgenic Res.

[6] Gustafsson E, Brakebusch C, Hietanen K, Fassler R (2001). Tie-1-directed expression of Cre recombinase in endothelial cells of embryoid bodies and transgenic mice. J Cell Sci.

[7] Kisanuki YY (2001). Tie2-Cre transgenic mice: a new model for endothelial cell-lineage analysis *in vivo*. Dev Biol.

[8] Motoike T, Markham DW, Rossant J, Sato TN (2003). Evidence for novel fate of Flk1+ progenitor: contribution to muscle lineage. Genesis.

[9] Alva JA (2006). VE-Cadherin-Cre-recombinase transgenic mouse: a tool for lineage analysis and gene deletion in endothelial cells. Dev Dyn.

[10] Newman PJ (1994). The role of PECAM-1 in vascular cell biology. Ann N Y Acad Sci.

[11] Privratsky JR, Newman PJ (2014). PECAM-1: regulator of endothelial junctional integrity. Cell Tissue Res.

[12] Kolossov E (2006). Engraftment of engineered ES cell-derived cardiomyocytes but not BM cells restores contractile function to the infarcted myocardium. J Exp Med.

[13] Schmidt A (2006). Endostatin influences endothelial morphology via the activated ERK1/2-kinase endothelial morphology and signal transduction. Microvasc Res.

[14] Schmidt A (2005). Endostatin down-regulates soluble guanylate cyclase (sGC) in endothelial cells *in vivo*: influence of endostatin on vascular endothelial growth factor (VEGF) signaling. Endothelium.

[15] Nagy A (1990). Embryonic stem cells alone are able to support fetal development in the mouse. Development.

[16] Wenzel D (2009). beta(2)-adrenoceptor antagonist ICI 118,551 decreases pulmonary vascular tone in mice via a G(i/o) protein/nitric oxide-coupled pathway. Hypertension.

[17] Herz, K. *et al*. Visualization of endothelial cell cycle dynamics in mouse using the Flt-1/eGFP-anillin system. *Angiogenesis* (2018). 10.1007/s10456-018-9601-1 29417260

[18] Kawamoto T (2003). Use of a new adhesive film for the preparation of multi-purpose fresh-frozen sections from hard tissues, whole-animals, insects and plants. Arch Histol Cytol.

[19] Wenzel D (2013). Endocannabinoid anandamide mediates hypoxic pulmonary vasoconstriction. Proc Natl Acad Sci USA.

[20] Vosen S (2016). Vascular Repair by Circumferential Cell Therapy Using Magnetic Nanoparticles and Tailored Magnets. ACS Nano.

[21] Malan D (2010). Endothelial beta1 integrins regulate sprouting and network formation during vascular development. Development.

[22] Vosen S (2016). Improvement of vascular function by magnetic nanoparticle-assisted circumferential gene transfer into the native endothelium. J Control Release.

[23] Redick SD, Bautch VL (1999). Developmental platelet endothelial cell adhesion molecule expression suggests multiple roles for a vascular adhesion molecule. Am J Pathol.

[24] Chatterjee S (2014). Artefacts in histopathology. J Oral Maxillofac Pathol.

[25] Drake CJ, Fleming PA (2000). Vasculogenesis in the day 6.5 to 9.5 mouse embryo. Blood.

[26] Baldwin HS (1994). Platelet endothelial cell adhesion molecule-1 (PECAM-1/CD31): alternatively spliced, functionally distinct isoforms expressed during mammalian cardiovascular development. Development.

[27] Kazemi S (2002). Differential role of bFGF and VEGF for vasculogenesis. Cell Physiol Biochem.

[28] Kearney JB, Kappas NC, Ellerstrom C, DiPaola FW, Bautch VL (2004). The VEGF receptor flt-1 (VEGFR-1) is a positive modulator of vascular sprout formation and branching morphogenesis. Blood.

[29] Zeng G (2007). Orientation of endothelial cell division is regulated by VEGF signaling during blood vessel formation. Blood.

[30] Dasgupta B, Dufour E, Mamdouh Z, Muller WA (2009). A novel and critical role for tyrosine 663 in platelet endothelial cell adhesion molecule-1 trafficking and transendothelial migration. J Immunol.

[31] DeLisser HM, Newman PJ, Albelda SM (1994). Molecular and functional aspects of PECAM-1/CD31. Immunol Today.

[32] Baker M (2011). Use of the mouse aortic ring assay to study angiogenesis. Nat Protoc.

[33] Nicosia RF (2009). The aortic ring model of angiogenesis: a quarter century of search and discovery. J Cell Mol Med.

