# Supplementary material for: PECAM/eGFP transgenic mice for monitoring of angiogenesis in health and disease
Source: Sci Rep. 2018 Dec 4;8:17582. doi: 10.1038/s41598-018-36039-2 (PMC6279819; doi:10.1038/s41598-018-36039-2)
Supplement: Supplementary file 1 — Data supplement [file 41598_2018_36039_MOESM1_ESM.pdf]

Data supplement

PECAM/eGFP transgenic mice for monitoring of angiogenesis in health and disease

Florian Winkler[1], Katia Herz[1], Sarah Rieck[1], Kenichi Kimura[1], Tianyuan Hu[1], Wilhelm Röll[2], Michael Hesse[1], Bernd K. Fleischmann[1], *Daniela Wenzel[1]

[1]Institute of Physiology I, Life&Brain Center, Medical Faculty, University of Bonn, Germany

[2]Department of Cardiac Surgery, Medical Faculty, University of Bonn, Germany

*Corresponding author:

Daniela Wenzel, MD

Institute of Physiology I, Life&Brain Center,

Medical Faculty, University of Bonn

Sigmund-Freud-Str. 25

53127 Bonn

Germany

Tel: 0049/228/6885/216

Fax: 0049/228/6885/201

Email: dwenzel@uni-bonn.de

Supplementary figures

Figure S1 Sketch of BAC recombination strategy. The eGFP reporter gene was cloned into the first exon of the PECAM gene within the PECAM BAC.

Figure S2 Endothelial-specific eGFP expression in PECAM/eGFP embryos at different developmental stages. (A-D) Fluorescence images of PECAM/eGFP embryos at E9.5 reveal strong eGFP expression in the developing vasculature when compared with littermates (A). The dotted white line (A) defines the surface of an $eGFP^-$ littermate. (B-D) $eGFP^+$ sprouts could be identified as brain vessels (BV, B), branchial arch (BA), second and third branchial arch artery (2nd, 3rd BAA), outflow tract of the heart (OFT), common atrial chamber (CAC), common ventricular chamber (CVC) (all C) as well as intersomitic vessels (ISV, D). (E-P) Sections of embryos at E15.5 reveal co-localization of eGFP expression (green) and PECAM staining (red) in the heart (E-G, note that heart has already been shown in Fig. 2 E), gut (H-J), lung (K-M) and paw (N-P). Green = native eGFP, red = autofluorescence (AF) (A), PECAM (F,G,I,J,L,M,O,P); bars = 1000 µm (A), 200 µm (B-G), 100 µm (H-J), 50 µm (K-P).

Figure S3 EGFP expression in different organs of PECAM/eGFP transgenic adult mice. Immunostainings demonstrate prominent and PECAM-specific eGFP expression in the vasculature of the uterus (A-C) and the gut (D-F). Green = native eGFP, red = PECAM, blue = Hoechst; bars = 25 µm (A-C), 50 µm (D-F).

Figure S4 EGFP expression in large vessels of flt-1/eGFP and PECAM/eGFP adult mice. (A) Fluorescence images of a flt-1/eGFP aorta (top) and a PECAM/eGFP aorta (bottom) illustrate differential eGFP expression in large arteries of both reporter gene models. (B-I) Immunostaining of sections from a flt-1/eGFP aorta (B-E) and a PECAM/eGFP aorta (F-I) reveal that the $PECAM^+$ endothelium (red) is preserved in both aortas. PECAM staining displays a perfect overlap with eGFP expression in PECAM/eGFP aortas. The white

rectangles in (D) and (H) define magnified regions in (E) and (I). (J-L) Immunofluorescence staining of sections from a PECAM/eGFP portal vein demonstrates eGFP expression also in venous endothelium. Green = native eGFP, red = PECAM, blue = Hoechst; bars = 1000 μm (A), 200 μm (B-D, F-H, J-L), 50 μm (E, I).

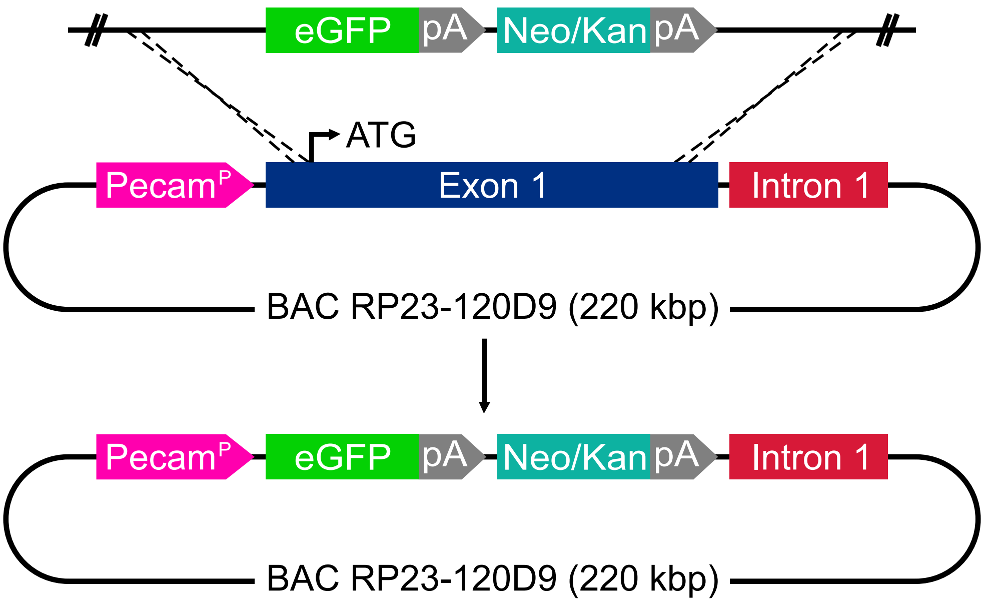
eGFP
pA
Neo/Kan
pA
ATG
Pecam P
Exon 1
Intron 1
BAC RP23-120D9 (220 kbp)
Pecam P
eGFP
pA
Neo/Kan
pA
Intron 1
BAC RP23-120D9 (220 kbp)

figure S1

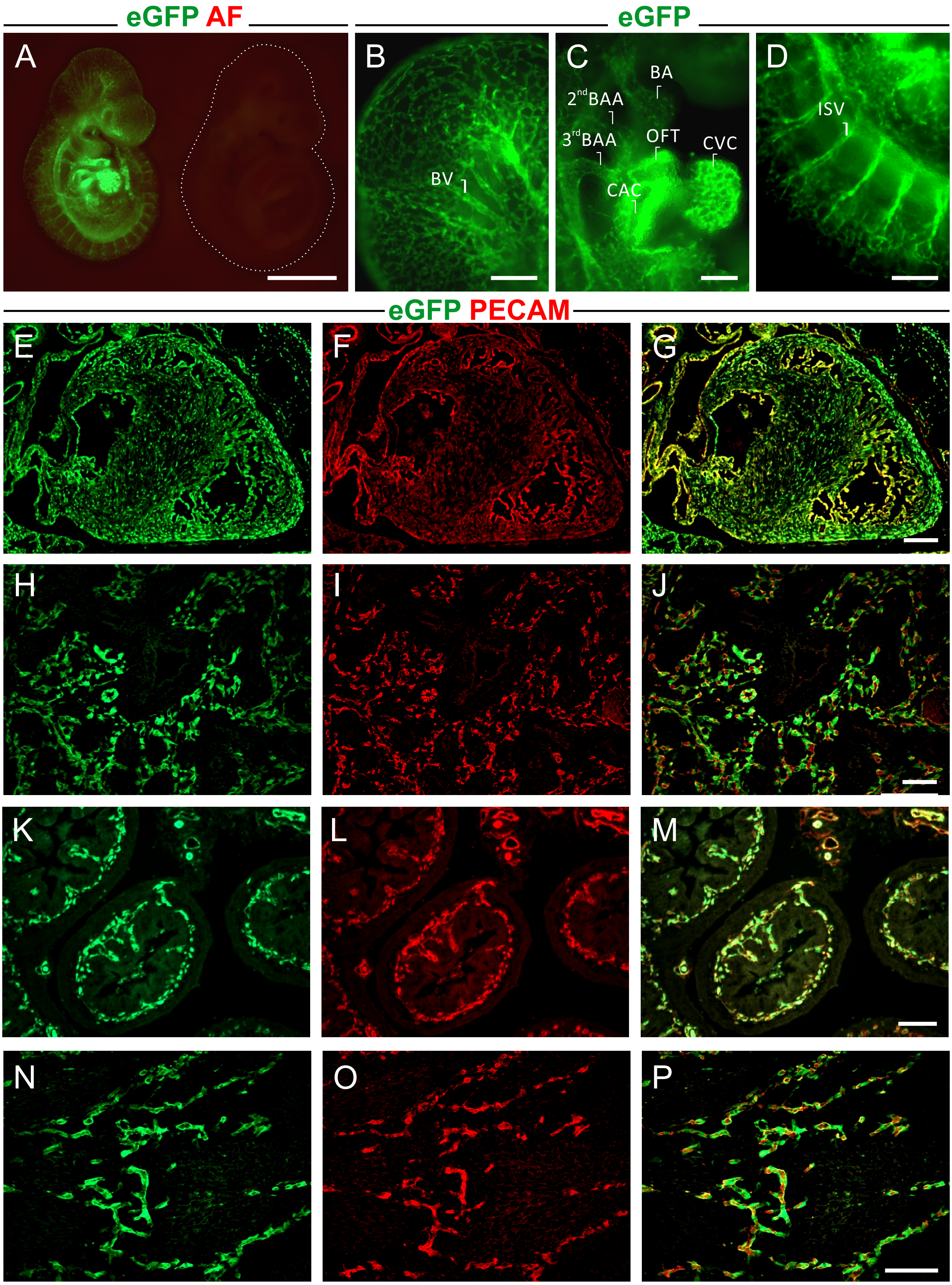

eGFP AF
eGFP
A
B
BV
C
BA
2nd BAA
3rd BAA
OFT
CVC
CAC
D
ISV
eGFP PECAM
E
F
G
H
I
J
K
L
M
N
O
P

figure S2

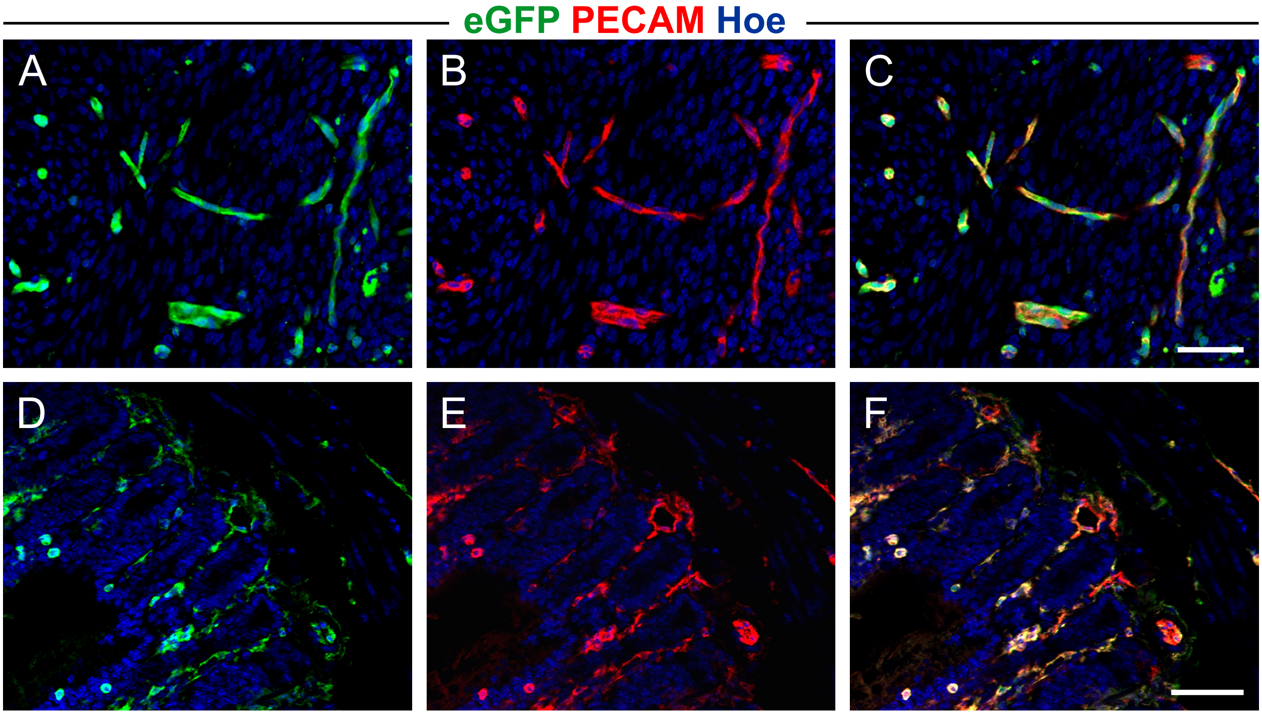

figure S3

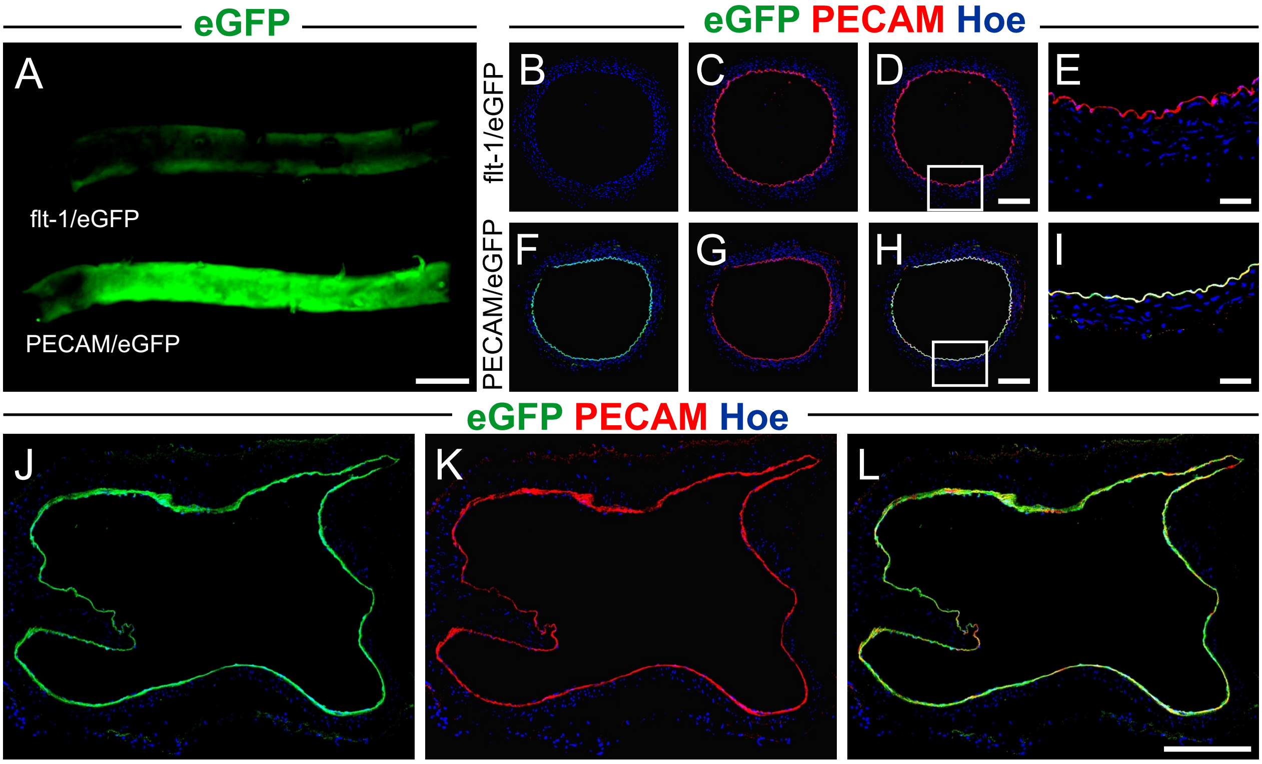
eGFP
eGFP PECAM Hoe
A
B
C
D
E
flt-1/eGFP
F
G
H
I
PECAM/eGFP
eGFP PECAM Hoe
J
K
L

figure S4
